# Hyperprolactinaemia with amisulpride

**DOI:** 10.4103/0019-5545.39761

**Published:** 2008

**Authors:** Rajnish Raj, Balwant Singh Sidhu

**Affiliations:** Department of Psychiatry, GMC and Rajindra Hospital, Patiala, India

**Keywords:** Amisulpride, ARIZONA Sexual Experience Scale (ASEX), galactorrhoea, hyperprolactinaemia

## Abstract

Amisulpride, a substituted benzamide derivative, is a second-generation antipsychotic that preferentially binds to D2/D3 receptors in limbic rather than striatal structures. High dosage preferentially antagonizes postsynaptic receptors, resulting in reduced dopamine transmission; and low dosage blocks presynaptic receptors, resulting in enhanced transmission. Hyperprolactinaemia may occur in patients receiving amisulpride at low dose of 50 mg/day and results in galactorrhoea, amenorrhea and sexual dysfunction. The symptom ameliorates on withdrawing the drug, switching to non-prolactin-elevating drugs, and timely management with dopamine agonist.

## INTRODUCTION

Psychosis is a disruptive mental state in which an individual struggles to distinguish the external world from his or her internally generated perceptions. Common symptoms of psychosis include hallucinations, delusions, and difficulty with thought organization. The humans' endeavor to solve the riddle of mental illnesses like schizophrenia and other psychotic disorders inspired them to venture into the vast land of molecules. A neuro-chemical approach affords the best explanation for the effectiveness of antipsychotic agents. The most important view is dopamine hypothesis. Dopamine hypothesis of schizophrenia was conceptualized by Matthysse.[1] Four dopaminergic tracts are important for understanding the actions of psychotic drugs: mesolimbic, mesocortical, nigrostriatal, and tuberoinfundibular systems. There are five types of dopamine receptors, with a total of more than 20 sub-variants; the dopamine hypothesis embraces only the D2 receptor. The clinical effectiveness of the drug occurs when about 60-70% of the dopamine D2 receptors are blocked.[2] Kapur and Seeman[3] have argued that what really makes atypical antipsychotics “atypical” is how fast they bind to and release from dopamine receptor. In contrast, therapeutic doses of clozapine usually are associated with lower levels of occupation of D2 receptors (40-50%) but higher (70-90%) levels of occupation of cortical 5 HT 2 A receptors.[4]

Amisulpride is a substituted benzamide derivative and a highly selective D2 and D3 receptor antagonist.[5] It has selective affinity for human D2 and D3 receptor subtypes *in vitro,* with a binding constant (ki) of about 3 nmol/l.[6] In *ex vivo* binding studies, amisulpride was twofold more selective for D3 receptors than for D2 receptors.[7] The atypical profile of an antipsychotic is linked to antagonism of both serotonin 5 HT2 A and D2- receptors; however, amisulpride lacks 5 HT 2A antagonism, and the atypicality may be explained by its preferential action on limbic D2/D3 receptors. The low dosage of amisulpride (< 10 mg/kg) preferentially blocks presynaptic D2/D3 receptors.[8] It induces a mean occupancy of 14% striatal D2 receptor.[9] The prolactin secretion is controlled by a complex mechanism, of which dopamine is the principal inhibitory component.[10] The effect on prolactin secretion is due to a blockade of pituitary action of the tuberoinfundibular dopaminergic system that projects from the arcuate nucleus of the hypothalamus to the median eminence. D2 receptors on mammotrophic cells in the anterior pituitary mediate the prolactin-inhibiting action of dopamine secreted at the median eminence into hypophyseal portal system. The increase in plasma prolactin levels is associated with D2 receptor antagonism.[11] Elevated levels of prolactin in females cause menstrual disturbances and galactorrhoea[12,13] and have been linked with disturbed sexual function in terms of desire, erections, orgasm in females and may even cause hypogonadism.[14,15] A case report is being highlighted having hyperprolactinaemia with a low dosage of the novel antipsychotic drug amisulpride.

## CASE

A middle-aged 43 years old woman, a B.Sc. degree holder, married, mother of one adolescent child and working as a staff nurse in the government sector, was brought by her husband to the Department of Psychiatry, Government Medical College and Rajindra Hospital, Patiala. The illness had begun some 5 years prior to this visit, when she started growing suspicious of her husband because he showed decreased interest in sex. She said that her husband was having an affair with another woman. When asked further about the affair, she said that her husband was involved with the neighbor's wife. She had not seen him in this woman's presence, nor of course did he admit to seeing her. She had vigilantly watched the neighbor's house frequently to spot him going in and out but unfortunately had not been there on the right occasions. Her husband denied the affair. He had never met the neighbor's wife nor had he entered her house. His wife repeatedly questioned him about the affair and become angry at his denials. He had found her searching his clothes, dresses, and several times following him. On further questioning, she admitted that she had searched her husband's purse and clothes for condoms. She added that she had also examined his underclothes for stains that might suggest recent intercourse. She could produce no evidence to the effect that he was having sexual relationship with another woman but was nevertheless convinced that they were engaged in a sexual liaison. Her childhood and adolescence were unremarkable. She graduated from college and worked successfully as a staff nurse in the government sector. She had been married for 17 years and had been happy until the last 5 years. However, she had always been mildly jealous of her husband when he was in the company of other women, although she had never previously accused him of infidelity. There was no relevant medical and psychiatric disorder. She was an introvert and had few friends. She was successful in her work but lacked confidence in social situations, especially with men. Except for her tendency to be jealous of her husband, she was proud of her daughter and was a good wife and mother. She was smartly dressed, appeared irritable in her relationship with her husband, but there was good rapport with the examiner. She sat quietly; and when asked questions, she talked coherently and with normal flow. She was well oriented with respect to time, person, and place. She admitted feelings of worry and anxiety because of her husband's affair. She firmly believed that her husband was having sexual relationship with the neighbor's wife, although she had never been able to find any firm proof of it and her husband always denied it. There were neither any signs of hallucinations nor of insight and reality contact broken. Physical examination and laboratory investigations revealed nothing abnormal. For about 5 years, she had experienced jealously, with a conviction that her husband was unfaithful. This belief was unshaken; her husband's denial and even lack of proof, for which she had searched frantically, had not changed her conviction. If one believed her husband's version of the story, then one would conclude that she had delusion. In the absence of hallucinations and organic cause and with duration of more than 3 months, the clinical picture fits well into the diagnosis of delusional disorder (F 22.0). Depressive symptoms may be present intermittently, provided that the delusion persists at times when there is no disturbance of mood. The delusional disorder may be specified as “jealous” type. On cross-sectional psychometric assessment, following were the scores on various scales before instituting the novel antipsychotic drug amisulpride: brief psychiatric rating scale (BPRS) score of more than 36 points; clinical global impression (CGI-S) score, 4; and global assessment of functioning scale (GAF) score, 27. After a written informed consent, on flexible dosing schedule, amisulpride 50 mg/day and lorazepam 2 mg were prescribed to be taken at night, with intention to treat; and periodic follow-up every two weeks was advised. In 2^nd^ week, she reported breast engorgement, galactorrhoea, decreased sexual desire, and sadness. Laboratory investigation, hemogram, urine test, electrolyte, renal and liver function tests, thyroid function, and FSH/LH were within normal limits; except prolactin level of 96.4 ng/ml measured on Tech-MEIA on AXSYM automated system, which was more than four times the normal range (1.39-24.20 ng/ml). BPRS showed 20% reduction in total score; Montgomery-Asberg depression rating scale (MADRS) score was 14; Arizona sexual experience scale (ASEX) score was 22, indicating clinically significant medication-induced sexual dysfunction; GAF score was 31; CGI-I score, 3; and CGI-efficacy score of 8. There was partial remission of psychotic symptoms - four-point increase in the global level of functioning, mild depression, and sexual dysfunction. The drug's side effect profile interferes with patient's functioning, and it outweighs the therapeutic effects. Hence amisulpride 50 mg/day was stopped, and she was over to olanzapine 15 mg/day, Amantadine 200 mg/day, and lorazepam 1 mg for the next two weeks. On 4^th^ week, prolactin levels came to baseline (22.3 ng/ml); there was 40% improvement in BPRS total score; MADRS score was 9; ASEX score was 14; CGI-I score was 3; and CGI-efficacy index score was 6, indicating improvement.

## DISCUSSION

Amisulpride is a substituted benzamide derivative and a highly selective presynaptic dopamine D2/D3 receptor blocker at low dose (50-100 mg/day), thereby enhancing dopamine transmission and possibly improving negative symptomatology; whereas higher doses (630-910 mg/day) antagonize postsynaptic D2/D3 receptors, thereby inhibiting dopamine transmission and possibly improving positive symptomatology.[9,16] Increase in plasma prolactin levels was associated with D2 receptor antagonism and found to be markedly greater with amisulpride than with other atypical agents (risperidone, olanzapine, and quetiapine) in a 2-week study in patients with schizophrenia.[11] Daytime hormonal changes produced by a single dose of amisulpride (20 or 100 mg intravenously) induce a fourfold to fivefold increase in prolactin area under the plasma concentration curve and requires regular monitoring of the patients.[17] Amisulpride has a potent prolactin-elevating effect, similar to conventional antipsychotic drugs. Hyperprolactinaemia has been reported in patients receiving amisulpride dosage of 50 mg/day as an augmentation to antidepressant therapy.[18] The case report highlights the acute antagonist effect of D2 receptor by amisulpride with a potent rise in prolactin levels causing galactorrhea and sexual dysfunction. However, the effect was rapidly reversible when the drug was discontinued;[19] as seen in this study. Further, to ameliorate the symptoms of psychosis, the patient required continuation of antipsychotic drugs. The approach was in line with that of the authors who treated patients with symptomatic hyperprolactinaemia, by switching to a non-prolactin-elevating drug (clozapine, olanzapine, quetiapine, aripiprazole, or ziprasidone).[19–21] The dopamine agonists (Amantadine, Cabergoline, and Bromocriptine) may be effective but have the potential to worsen psychosis.[20–22] There is partial change in the psychopathology during the first two weeks of trial of amisulpride 50 mg/day, but the side effect of hyperprolactinaemia outweighs the therapeutic benefit.

This difference could be attributed to the genetic variability[23] and requires further studies. Hence the utility of a designer drug in maximizing efficacy and receptor selectivity and achieving better tolerability is far from reality and is yet to be conceived - a field still open for research.
